# Contribution of vitamin B 6 deficiency to anemia in children on regular hemodialysis

**DOI:** 10.1186/s12887-025-05386-1

**Published:** 2025-01-27

**Authors:** Fatma Atia, Hafez Bazaraa, Yara Abdelatty, Sarah Lotfy, Eman Eryan

**Affiliations:** 1 Department of Pediatrics, Center of Pediatric Nephrology and Transplantation (CPNT), Kasr Al Ainy Faculty of Medicine, Cairo University, Cairo, Egypt; 2 Department of Clinical & Chemical Pathology, Kasr Al Ainy Faculty of Medicine, Cairo University, Cairo, Egypt; 3 Cairo University Children Hospital, Kasr Al Ainy, Cairo, Egypt

**Keywords:** Vitamin B6, Children, Hemodialysis, Anemia, ESKD

## Abstract

**Background:**

Anemia is prevalent among pediatric patients diagnosed with end-stage kidney disease (ESKD). In addition, erythropoiesis-stimulating agents (ESA) and iron supplementation are considered the cornerstones in the management of anemia. However, a significant proportion of patients remain anemic. Vitamin B6 plays a vital role in the biosynthesis of heme and may be deficient in children with chronic kidney disease (CKD), particularly those on regular hemodialysis (HD). We aimed to evaluate serum vitamin B6 concentration in those children and determine its correlation with anemia indices.

**Methods:**

The current cross-sectional study included 39 children on regular HD and 43 healthy controls. Clinical data were collected, including anthropometric measurements, blood pressure, iron, and erythropoietin therapy. Laboratory investigations included hemoglobin (Hb) indices, iron profile, and vitamin B6 level.

**Results:**

The median Hb level in our cohort was 10.5 g/dL, and nine patients had Hb levels at or above the target Hb of 11 g/dl. They had a median Serum Vitamin B6 concentration of 28.2 ng/ml (IQR = 22.8–52.9) compared to a median of 27.5 ng/ml(IQR = 20–34) in controls, with no substantial differences between both groups. There was no statistically significant correlation between vitamin B6 and Hb levels or erythropoietin dose.

**Conclusion:**

It is evident that the majority of our patients did not meet the desired threshold for anemia control. However, it is noteworthy that the average hemoglobin (Hb) level approached the intended target. The incidence of Vitamin B6 deficiency was not found to be statistically significant within our study population. Therefore, we could not establish a correlation between vitamin B6 deficiency and anemia in children on HD.

## Background

Anemia and its management pose a significant challenge for clinicians in the context of pediatric CKD, which is characterized by the presence of multiple comorbidities [1]. It is a substantial factor in determining the quality of life, fatigue, and morbidity among these patients [2, 3].

Vitamin B6 is a crucial coenzyme with rate-limiting properties that significantly contribute to the biosynthesis of heme [4]. Therefore, a deficiency in vitamin B6 has the potential to induce anemia. The anemia typically presents as microcytic, hypochromic, and sideroblastic [2]. The occurrence of anemia has been reported in cases with CKD, especially those who require dialysis and who received an erythropoiesis-stimulating agent (ESA) [4].

Several factors were reported to induce vitamin B6 deficiency in adult individuals receiving chronic dialysis, including decreased dietary intake, decreased absorption, altered pyridoxal metabolism, increased dialysis losses, and drug interactions [3].

According to the Kidney Disease Outcomes Quality Initiative (K/DOQI) of the National Kidney Foundation, it has been noted that adult renal patients undergoing maintenance dialysis exhibit decreased vitamin B6 levels. There is a scarcity of data pertaining to the documentation of vitamin B6 levels in pediatric patients undergoing dialysis. K/DOQI suggested that adult individuals receiving dialysis should take a daily supplement of pyridoxine-HCL (10 mg) [4].

The recent micronutrient guidelines from the European Society for Clinical Nutrition and Metabolism (ESPEN) have emphasized the need to increase the consumption of vitamins B6, B3, B1, and folic acid due to a heightened risk of inadequate levels of these nutrients among CKD patients [5].

Our primary objective is to evaluate serum vitamin B6 status in those children and determine its correlation with anemia indices.

## Methods

### Study design and population

The present cross-sectional study involved 39 ESKD children (on regular HD) in addition to 43 healthy controls matched in terms of sex and age. Patients diagnosed with ESKD were recruited from the Center of Pediatric Nephrology and Transplantation at Cairo University Children’s Hospital. The control group consisted of individuals attending outpatient clinics for routine checkups or follow-up visits after receiving treatment for minor medical conditions, the study period was from July 2022 to April 2023.

The inclusion criteria were age (from 1 to 14 ) years, ESKD of any etiology, and patients receiving HD for at least six months. We excluded the participants who were on medications that could potentially affect vitamin B 6 status (phenytoin -valproic acid – carbamazepine).

### Ethical consideration

The study was conducted in accordance with the declaration of Helsinki. In addition, the institutional review board approved the study procedures [Approval number is MS-96-2022]. Parents or eligible guardians provided informed consent. The data privacy of all subjects was guaranteed.

### Sample size

The study involved 39 cases and 43 controls. This sample size is consistent with assumed prevalence rates of vitamin B6 deficiency in 20% of controls and 50% of cases (*N* = 38 in each group), and a power of 80% at an alpha error rate of 0.05.

### Clinical assessment

Full clinical assessment included age, primary renal disease, anthropometry, blood pressure, current medications in the past three months, and any reported blood loss, transfusion, erythropoietin dose, and iron therapy.

### Routine laboratory tests

The average predialysis Hb concentration over the past three months was obtained for each patient and also the current hemoglobin. We defined anemia according to Kidney disease improving global outcome(KDIGO 2012) guidelines as following: if Hb concentration is 11.0 g/dl in children 0.5–5 years, 11.5 g/dl in children 5–12 years, and 12.0 g/dl in children 12–15 years [6].and we used for sub group analysis Hb concentration of 11 g/dl or above [7].

#### Vitamin B6 measurement

Pyridoxal phosphate(PLP) was measured in predialysis serum samples using double-antibody sandwich enzyme linked immunoadsorbant essay (ELISA) (test kit from SunRed Biological technology- catalog No 201-12-1540).

### Statistical analysis

Data analysis and coding were done utilizing the 28th version of the SPSS software (IBM Corp.-Armonk-NY-USA). Parametric data were expressed using standard deviations and means, whereas Categorical data were expressed as number (and proportion). Comparisons between quantitative variables were done using Mann-Whitney and non-parametric Kruskal-Wallis tests. Categorical data were compared utilizing the Chi-square (X2) test. The Spearman correlation coefficient [6] revealed correlations between quantitative variables. P-values < 0.05 were considered statistically significant.

## Results

A total of 39 children were recruited in the study, with a mean age of 9.23 (± 2.88) years; 59% were females. Of the cohort, 26 patients (66.7%) were offsprings of consanguineous parents, and 17.9% had a positive family history of CKD. The most common etiology of ESKD was reflux nephropathy in 25.6% of the cases. Twenty-eight patients (71.8%) fell below − 2 standard deviation (SD) score for weight, 29 patients (74.4%) for height, and 14 patients (35.9%) for body mass index (BMI). All patients were hypertensive, receiving an average of two antihypertensive medications. Blood pressure was controlled on medications in 27 cases (69.2%). The median duration of dialysis was one year, ranging between 6 months and nine years, all of them underwent 3 sessions of hemodialysis per week with blood flow rate ranging from 120–130/min and the median Kt/v was 1.3, the baseline characteristics are depicted in Table (1).

**Table 1:** The basic clinical and laboratory data of the study group (*n* = 39)

Characteristics	Count	Frequency
Age (years)	9	3–14
Gender (Female)	23	59%
Primary disease		
Unknown	10	25.6%
Reflux nephropathy	10	25.6%
Primary hyperoxaluria	5	12.8%
FSGS	4	10.3%
PKD	3	7.7%
Nephronophthisis	2	5.1%
Bardet Biedel	1	2.6%
Caroli disease	1	2.6%
Cystinosis	1	2.6%
HUS	1	2.6%
Joubert’s syndrome	1	2.6%
Weight below − 2 z-score	28	71.8%
Height below − 2 z -score	29	74.4%
**Parameter**	Median	IQR
Average Hemoglobin in the last 3 months	10.5	9–12
Current Hemoglobin (g/dl)	10.2	9–11
MCV (fl.)	86	83–90
Iron (umol/l)	61	37–87
Ferritin (ug/l)	1184	766–1796
TIBC (ug/dl)	184	163–233
TSAT (%)	30	4–90

Median and range; FSGS: focal segmental glomerulosclerosis; PKD: polycystic kidney disease; HUS: hemolytic uremic syndrome; MCV: mean corpuscular volume; TIBC: total iron binding capacity; TSAT: transferrin saturation

Regarding anemia, the average Hb level within the past three months had a median of 10.5 g/dl (IQR = 9–12 g/dl) and the current Hb concentration had a median of 10.2 g/dl (IQR = 9–11 g/dl). In contrast, most patients (61.5%) did not require any blood transfusion in the last three months, 30.8% received a single blood transfusion, and 7.7% received two transfusions.

The majority of patients (89.7%) were on iron therapy either oral iron with a dose of 3 mg/kg/day or intravenous iron (ferrous glucogonate) with a maintenance dose of 1 mg/kg/week guided by the iron studies, and 97% of the patients received beta-erythropoietin with median dose of 375 IU/kg/week (IQR = 272–500).

We compared patients who had anemia (*n* = 18) with those who did not have anemia (*n* = 6) after excluding patients who received blood transfusion (*n* = 15). There was no significant difference between the levels of Vitamin B6, as shown in Table (2).

**Table 2:** Comparison between anemia and non-anemia groups regarding hemoglobin indices and vitamin B6 level (*n* = 24)

Median (IOR)	Non -anemia group (*n* = 6)	Anemia group (*n* = 18)	*p*-value
Age /years	10.5 (8.5–14)	9.5 (7.5–12)	0.343
MCV (fl.)	86.5 (85–90)	86.5 (82.3–90.3)	0.721
MCH (pg)	28.5 (26.5–30.5)	27 (24.7–29)	0.177
Erythropoietin dose (IU/kg/week)	288 (121–549)	308 (245–383)	0.871
Vitamin B6 level	33.8 (18.1–69.5)	26.5 (20-52.3)	0.770

15 cases were excluded as they received blood transfusion

IQR: interquartile range; MCV: mean corpuscular volume; MCH: mean corpuscular hemoglobin; IU: international unit; kg: kilogram

We used a hemoglobin concentration of 11 g /dl as a target level for comparison knowing that most of our patients were on ESA treatment, then we did subgroup analysis for those patients with hemoglobin at this level or above it and had no history of blood transfusion; we found nine patients (23%) with Hb level of 11 g/dl. Four of these nine patients had serum vitamin B6 concentrations above the normal range (52,61.3,78,99.5 ng/ml).

The median level for vitamin B6 in our study group was 28.2 ng/ml (IQR = 22.8–52.9), and the median serum vitamin B6 level for the control group was 27.5 ng/ml (IQR = 20–34) with no significant difference between both groups (p value = 0.207); where two patients fell below the normal range (5–50 ng/ml). These two patients had Hb levels of 11.1 g/dL and 10.9 g/dL with no history of blood transfusion in the past six months.

A total of 3 patients in our study group were on oral vitamin B6 supplements (dose = 5-15 mg/kg/day, tab = 50 mg pyridoxine) as a treatment for primary hyperoxaluria. Their serum levels of vitamin B6 were (24, 25, 149) ng/ml. They did not fulfill the target Hb level within the last three months compared to other patients (P value = 0.024). One of the patients received a blood transfusion once during the last three months.

Table (3) shows no marked association between vitamin B6 level and other parameters of anemia, duration of dialysis, erythropoietin dose, or the frequency of blood transfusion.

**Table 3:** Relation of vitamin B6 levels to anemia indices, duration of dialysis, erythropoietin dose, and frequency of blood transfusion

		Serum Vitamin B6 level			
		Correlation Coefficient*	*P* value		**N**
Age/years		0.208	0.204		39
Duration of dialysis in years		0.145	0.380		39
Erythropoietin dose/week		0.023	0.888		39
Average Hemoglobin in the last 3 months		-0.025-	0.879		39
Current Hemoglobin concentration		-0.103-	0.532		39
MCV		0.064	0.700		39
MCH		-0.127-	0.440		39
Hct		-0.171-	0.298		39
Iron		-0.079-	0.632		39
Ferritin		-0.108-	0.511		39
TIBC		-0.068-	0.681		39
		**Median**	**Minimum**	**Maximum**	**P value ¶¶**
Number of blood transfusions in the last 3 months	2	23.40	18.90	26.60	0.133
	1	43.30	23.30	149.00	
	none	27.90	2.30	142.00	
Fulfillment of target Hemoglobin in the last 3 months	yes	36.50	2.80	99.50	0.620
	no	24.20	2.30	149.00	

MCV; mean corpuscular volume, MCH; mean corpuscular hemoglobin, Hct; hematocrit, TIBC; total iron binding capacity

*spearman non-parametric correlation

¶¶Chi-square test

## Discussion

We examined the correlation between serum vitamin B6 concentration and various indicators of anemia, including Hb indices, iron profile, and erythropoietin dose.

In our study, we found that Vitamin B6 deficiency did not pose a significant concern, as the median serum vitamin B6 level for the study group was 28.2(ng/ml). The median serum vitamin B6 level for the control group was 27.5 (ng/ml), with no significant difference found between both groups (*P* = 0.207). This finding is consistent with a study on children on regular dialysis that reported no deficiency [4]. Only two patients (5%) of the study group had deficient levels of vitamin B6, similar to another study that found vitamin B6 deficiency only in 2.5% of the included patients [8]. Similarly, another study found blood levels of vitamin B were normal in children receiving dialysis, indicating adequate status [9]. This finding contradicts different studies that found vitamin B6 deficiency ranges between 24 and 56% [10–12]. The difference between these studies could be explained by a discrepancy in the inclusion criteria, whether they included or not the patients on Vitamin B 6 supplements.

Despite the fact that vitamin B6 is crucial for anemia control, by correlating serum levels of vitamin B6 in the study group with Hb indices, serum iron, ferritin, erythropoietin dose, and the need for blood transfusion, we found no significant correlation between vit B6 level and any of them. This finding contradicts the results of a study conducted on adults undergoing regular dialysis, who were administered a daily dosage of 10 mg of pyridoxin [13]. Conversely, one of the two patients with vitamin B6 deficiency had an Hb level of 11.1 g/dl, in contrast to several reports that relate vitamin B6 deficiency to the development of anemia [1, 2, 14].

On the other side; it was observed that only nine individuals exhibited Hb levels equal to or exceeding 11 g/dL. 45% of these nine patients displayed elevated levels of vitamin B6. The evidence presented in our cohort does not strongly support a correlation between anemia control and the higher level of vitamin B6;Perhaps for optimal health, a higher status is needed.

Lack of evidence could be explained by lack of established guidelines for monitoring of vitamin B 6 in hemodialysis patients [15] and the levels of vitamin B6 measured in this study were of adult references.

Although the Kidney Disease: Improving Global Outcomes (KDIGO) international guidelines recommend a target Hb range of 11 to 12 g/dl for children with CKD [7], the findings of our study indicate that a significant majority (75%) of the patients failed to attain this target Hb level. Potential contributing factors could include malnutrition, as a significant number of our patients exhibit weight and height measurements below − 2 SD. Another possible factor is hyperphosphatemia, with a mean phosphorus level of 6.04 ± 2.28 mg/dl, as supported by a previous study that identified a correlation between anemia and hyperphosphatemia [16].

### Limitations

Firstly, understandably due to the rarity of the pediatric CKD5D, the cohort is small.

These data may not be generalizable to the greater pediatric CKD5HD population. The prevalence of severe anemia within our cohort was relatively low, and only two patients exhibited a deficiency in Vitamin B6. Therefore, we could not establish a correlation between them. Furthermore, a comprehensive evaluation of the individual’s nutritional background, specifically about factors that could potentially influence vitamin B6 levels, and measures of folate, Vitamin B12, and l- carnitine which would be important confounding nutrients that influence the primary outcome of anemia and ESA requirement were not conducted.Lastly, the intracellular concentrations are more reliable in patients with chronic diseases, we used ELISA based assessment of vitamin B6 in serum samples because of its more feasibility & less cost than the other methods such as HPLC or measuring the activity of red cell aspartate (or alanine) aminotransferase and its activation coeffcient on incubation with PLP.

## Conclusion

Our study did not provide evidence to support the association between vitamin B6 deficiency and anemia in children undergoing hemodialysis. However, it is observed that patients with well-managed anemia tend to exhibit elevated levels of vitamin B6, although this correlation lacks statistical significance.

To the best of our knowledge, this is the first study that attempted to correlate anemia and vitamin B6 levels in children on regular HD. We recommend further studies correlating high vitamin B6 and its impact on anemia control in children on regular HD.

## Data Availability

The datasets generated during and/or analyzed during the current study are available from the corresponding author on reasonable request.
